# Matrix metalloproteinase MMP9 maintains epithelial barrier function and preserves mucosal lining in colitis associated cancer

**DOI:** 10.18632/oncotarget.21841

**Published:** 2017-10-17

**Authors:** Adani Pujada, Lewins Walter, Aashka Patel, Tien Anh Bui, Zhan Zhang, Yuchen Zhang, Timothy Luke Denning, Pallavi Garg

**Affiliations:** ^1^ Institute for Biomedical Sciences, Georgia State University, Atlanta, GA, USA; ^2^ Department of Biology, Georgia State University, Atlanta, GA, USA

**Keywords:** colitis associated cancer, microbiota, MMP9, IL-22, A. muciniphila

## Abstract

In colitis associated cancer (CAC), chronic inflammation exposes the epithelial mucosal defensive lining to inflammatory mediators such as cytokines and anti-microbial peptides (AMPs) causing the dysbiosis of microbiota population and the dysregulation of immune response. Matrix Metalloproteinases (MMPs) are zinc dependent endopeptidases which mediate inflammation, tissue remodeling, and carcinogenesis. MMP9 is undetectable in healthy tissue, although highly upregulated during inflammation and cancer. We have previously shown that MMP9 plays a protective role in CAC opposite to its conventional role of acute inflammation and cancer mediator. In this study, we investigated the mechanistic role of MMP9 in preserving the epithelial mucosal integrity to suppress the progression of tumor microenvironment in CAC. We used transgenic mice constitutively expressing MMP9 in colonic epithelium (TgM9) as an *in vivo* model and intestinal cell line CaCo2BBE as an *in vitro* model. We induced CAC with three cycles of dextran sodium sulfate (DSS). We observed that MMP9 expression in colonic epithelium maintains the microbiota. We also observed that MMP9 mediates pro-inflammatory cytokine levels and AMPs but suppresses IL-22 resulting in lower levels of REG3-g and S100A8 AMPs. We also found that MMP9 maintains an efficient barrier function and the integrity of tight junctions. We also observed increased levels of mucin and intestinal trefoil factor among TgM9 mice in CAC. We also found that MMP9 expressing CaCo2BBE cells had increased expressions of EGFR and nuclear transcription factor- specificity protein 1 (Sp1). These data imply that MMP9 acts as a tumor suppressor in CAC by sustaining the epithelial mucosal integrity due to the activation of EGFR-Sp1 signaling pathway.

## INTRODUCTION

Inflammatory bowel disease (IBD) is an autoimmune disease. Repeated flares of IBD cause chronic inflammation in the gastrointestinal (GI) tract [1]. Ulcerative colitis (UC) and Crohn’s Disease (CD) are the two forms of IBD. Both, UC and CD, present similar symptoms, however, the relative affected areas and the progression of inflammation are very different. CD affects any area of the GI tract while UC affects the colon and the rectum causing inflammation and ulcers either in the segments or in the entire colon. Inflammatory lesions, caused by UC, are generally in the top layer of the colon [2]. Patients with severe UC have deep mucosal ulcerations. Chronic UC increases the risk of colitis associated cancer (CAC), a type of colon cancer driven by continuous exposure to inflammation. Therefore, CAC is uniquely and significantly different than sporadic colon cancer (CRC). UC increases the risk of CAC by up to 18-20% while CD up to 8% after the onset of the disease [3].

The GI epithelial-mucosal lining acts as an immune surveillance system via two barriers- external physical barrier and functional immunological barrier [4]. These barriers together protect against luminal insults such as invading environmental pathogens, toxins, dietary and microbial/viral peptides. Integrity of the epithelial mucosal lining is maintained by the paracellular space between the two adjacent epithelial cells, which is sealed by the tight junction (TJs) proteins. TJs maintain the flow of ions and small peptides between the lumen and the epithelial-mucosal tissues [4]. Epithelial mucosal lining harbors a population of over 1000 bacterial species (the gut microbiota) which co-habit in the small intestine and colon [5]. Gut microbiota, TJs and immune cells are equally important in homeostatic maintenance of the epithelial-mucosal immune system. The dysbiosis of the gut microbiota, dysregulation of TJs and/or misbalance of immune cells initiate the development of autoimmune diseases or cancer [6, 7].

MMPs are a large family of calcium-dependent, zinc-containing endopeptidases. They are classified as: collagenases, gelatinases, stromelysins, and matrilysins based on their ability to cleave extra cellular matrix (ECM) substrates [8, 9]. They are classified by their membrane type and their MMP number has been assigned in order of discovery. MMPs are secreted by cells to initiate remodeling by degradation of the extracellular matrix. The majority of MMPs are upregulated in response of pro-inflammatory cytokines, cell-ECM, or cell-cell interactions. They are also involved in adhesion and migration of the leukocytes from the blood vessels as an inflammatory response [9, 10]. Among all MMPs, MMP9 is unique for being involved in activating several signaling molecules and pathways during inflammation and cancer, despite being inactive in normal tissues [11–16]. MMP9 is one of the most studied MMPs as an IBD mediator and it is commonly secreted by epithelial cells, immune cells, neutrophils and sometimes by macrophages during inflammation [10, 16, 17].

We and others have previously shown that MMP9 is a mediator of acute colitis [11, 14, 18, 19] and colorectal cancer [20–22]. We have shown that MMP9 has a contrasting protective role in CAC in the setting of chronic inflammation and, acts as a tumor suppressor in CAC [12, 13, 15, 23]. Considering the fact that GI tract is mainly constituted of epithelial cells and colon cancer has epithelial origin, we have generated transgenic mice ‘Tg-villin-MMP9 (TgM9)’ that constitutively expresses MMP9 under villin promoter (TgM9) [14, 15]. The aim of this study is to investigate the mechanism by which MMP9 maintains the epithelial-mucosal integrity in CAC, as the ‘extrinsic mechanistic pathway’. Chronic inflammation drives the ‘extrinsic mechanistic pathway’ by triggering the activation of several signaling pathways. This happens due to the release of various biomolecules such as cytokines, chemokines and growth factors as the first response of the cell against the inflammation. Therefore, chronic inflammation is considered to be the seventh hallmark of the cancer [24].

## RESULTS

### MMP9 in colonic epithelium attenuates microbiota depletion in CAC

**Figure 1:**
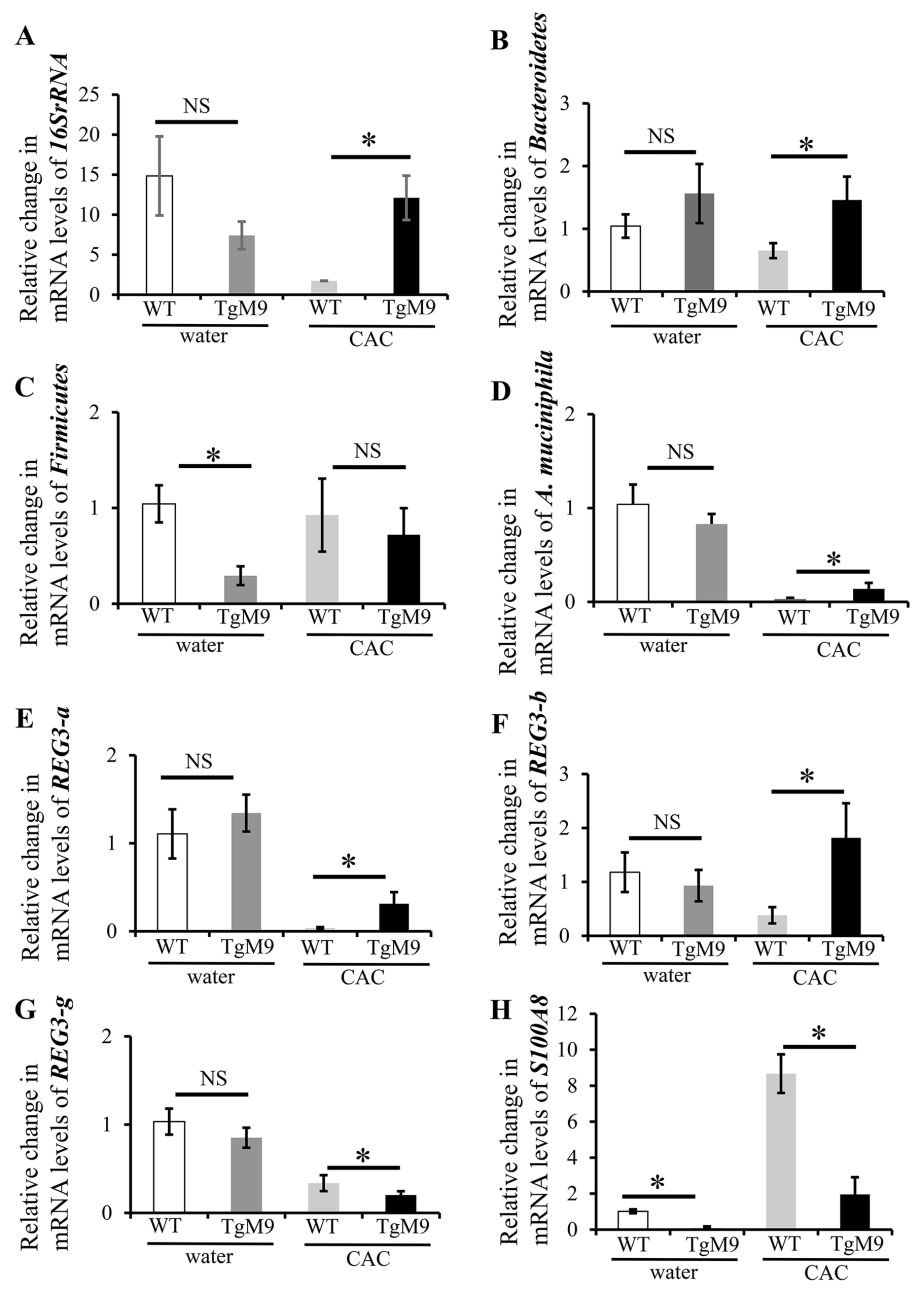
MMP9 in colonic epithelium attenuates microbiota depletion in CAC Bar graph representation of the QPCR analyses of different phyla of microbiota at mRNA levels in colonic mucosal strippings from WT and TgM9 mice treated with water or 3 cycles of 3% DSS. Relative mRNA expression levels of **(A)** universal bacteria, *16SrRNA* **(B)** *Bacteroidetes* **(C)** *Firmicutes* **(D)** *A. muciniphila* **(E)** *REG3-a* **(F)** *REG3-b* **(G)** *REG3-g* and **(H)** *S1008A*. NS means non-significant. Each bar represents mean ± S.E., *p< 0.05.

Microbiota imbalance is critical in triggering intestinal inflammation as well as determining the susceptibility to cancer. We investigated the role of MMP9 in maintaining the microbiota population in colon by QPCR. Figure 1A and 1B show that mRNA levels for *16SrRNA* (universal bacteria) and *Bacteroidete*s were significantly increased in TgM9 mice (12.10±2.77 fold and 1.46±0.37 fold) compared to wild type littermates (WT) (1.73±0.03 and 0.65±0.12) in CAC. In fact, mRNA levels of *Akkermansia muciniphila* (Figure 1D) were also higher in TgM9 mice (0.14±0.06 fold) compared to WT (0.03±0.01) mice in CAC. Interestingly, mRNA levels of *Firmicutes* (Figure 1C) were lower among TgM9 mice (0.72±0.28) compared to WT (0.93±0.38) mice in CAC. Interestingly, TgM9 exposed to water/ without CAC had lower mRNA levels for *16SrRNA* (Figure 1A), *Firmicutes* (Figure 1C) and *A. muciniphila* (Figure 1D) but higher *Bacteroidete*s (Figure 1B) compared to WT mice control group.

Antimicrobial proteins (AMPs) are integral component of innate immunity and are produced by the epithelial surface in response to invading pathogens, enteric microbiota and other luminal insults [25]. The regenerating islet-derived (REG) proteins are highly expressed in IBD related colonic inflammation [26]. REG family members from the same species are highly conserved. Our QPCR data (Figure 1E and 1F) showed a significant increase in the mRNA levels of *REG3-a* (0.31±0.13 fold) and *REG3-b* (1.82±0.64 fold) compared to WT mice (0.04±0.01 and 0.38±0.15) in CAC. Interestingly there was a significant decrease (Figure 1G) in the mRNA levels of *REG3-g* (0.20±0.04) among TgM9 mice compared to WT mice (0.34±0.09 fold more) in CAC. However, mRNA levels of *REG3-a*, *REG3-b* and *REG3-g* (Figures 1E-1G) were comparable between TgM9 and WT mice both exposed to water/ without CAC.

Damage-associated molecular pattern (DAMP) molecules such as S100A8 and S100A9, are also crucial in mediating inflammation. They are equally important in modulating tumor growth and metastasis [27]. Interestingly, we also observed (Figure 1H) a significant decrease in the mRNA levels of *S100A8* (1.96±0.95) among TgM9 mice compared to WT mice (8.67±1.07) in CAC. Remarkably, mRNA levels of *S100A8* (Figure 1H) among TgM9 were also significantly lower compared to WT mice both exposed to water/without CAC.

These data together indicate that in CAC the constitutive expression of MMP9 in colonic epithelium maintains microbiome by keeping a check on the pro-inflammatory AMPs and DAMPs molecules.

### MMP9 modulates cytokine levels in CAC

Cytokines released by immune cells are the first defensive response against an inflammatory insult. However, if the inflammation continues to be chronic, cytokines start nurturing the tumor microenvironment and favors tumor progression [28, 29].

**Figure 2:**
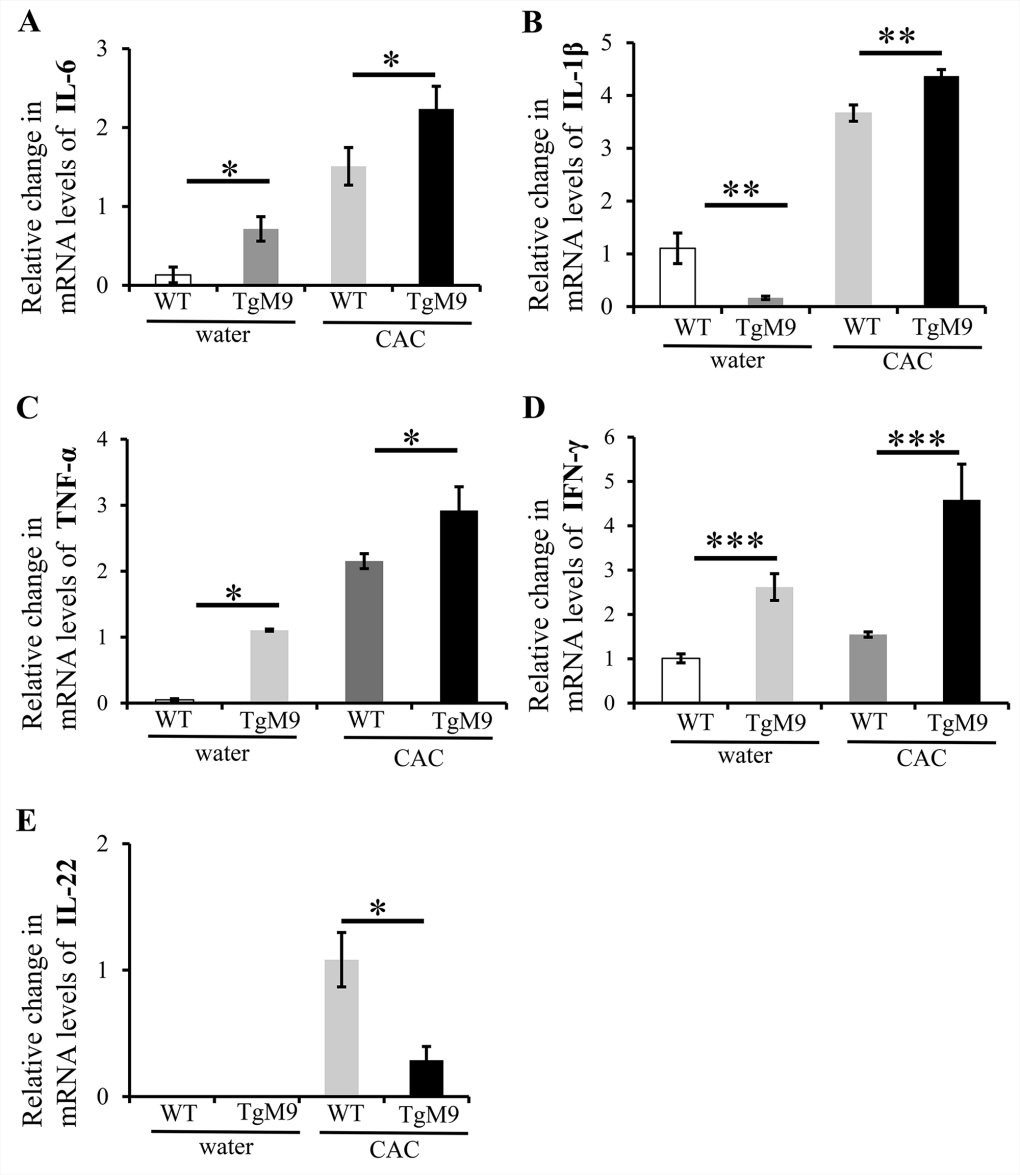
MMP9 modulates cytokine levels in CAC Bar graph representation of the QPCR analyses of mRNA levels of cytokines in colonic mucosal strippings from WT and TgM9 mice treated with water or 3 cycles of 3% DSS. Relative mRNA expression levels of the cytokines **(A)** IL-6 **(B)** IL-1β **(C)** TNF-α **(D)** IFN-γ and **(E)** IL-22 are shown. Each bar represents mean ± S.E., *p< 0.05, **p< 0.005, ***p< 0.0005.

We investigated the expression levels of the cytokines which are critical in mediating the progression of chronic inflammation to colon cancer by utilizing QPCR. We observed that mRNA levels of interleukin *(IL)-6* (2.24±0.28 fold), *IL-1β* (4.37±0.12 fold), tumor necrosis factor *(TNF)-α* (2.9±0.36 fold) and interferon *(IFN)-γ* (4.59±0.8 fold) were significantly increased among TgM9 mice compared to WT mice (1.51±0.24, 3.67±0.67, 2.15±0.11, and 1.55±0.06 respectively) in CAC (Figure 2A-2D respectively). Interestingly, the mRNA levels of *IL-22* (Figure 2E) were significantly lower among TgM9 mice (0.29±0.11) compared to WT mice (1.08±0.21) in CAC. TgM9 mice also showed significantly increased levels of mRNA for *IL-6*, *TNF-α* and *IFN-γ* (Figure 2A, 2C and 2D respectively) except for *IL-1β* (Figure 2B) compared to WT mice, both exposed to water/ without CAC. There was negligible expression of mRNA for *IL-22* in TgM9 and WT mice both exposed to water/ without CAC (Figure 2E). This implies that MMP9 modulates cytokine levels in CAC.

### MMP9 maintains colonic epithelial barrier function and TJ integrity in CAC

**Figure 3:**
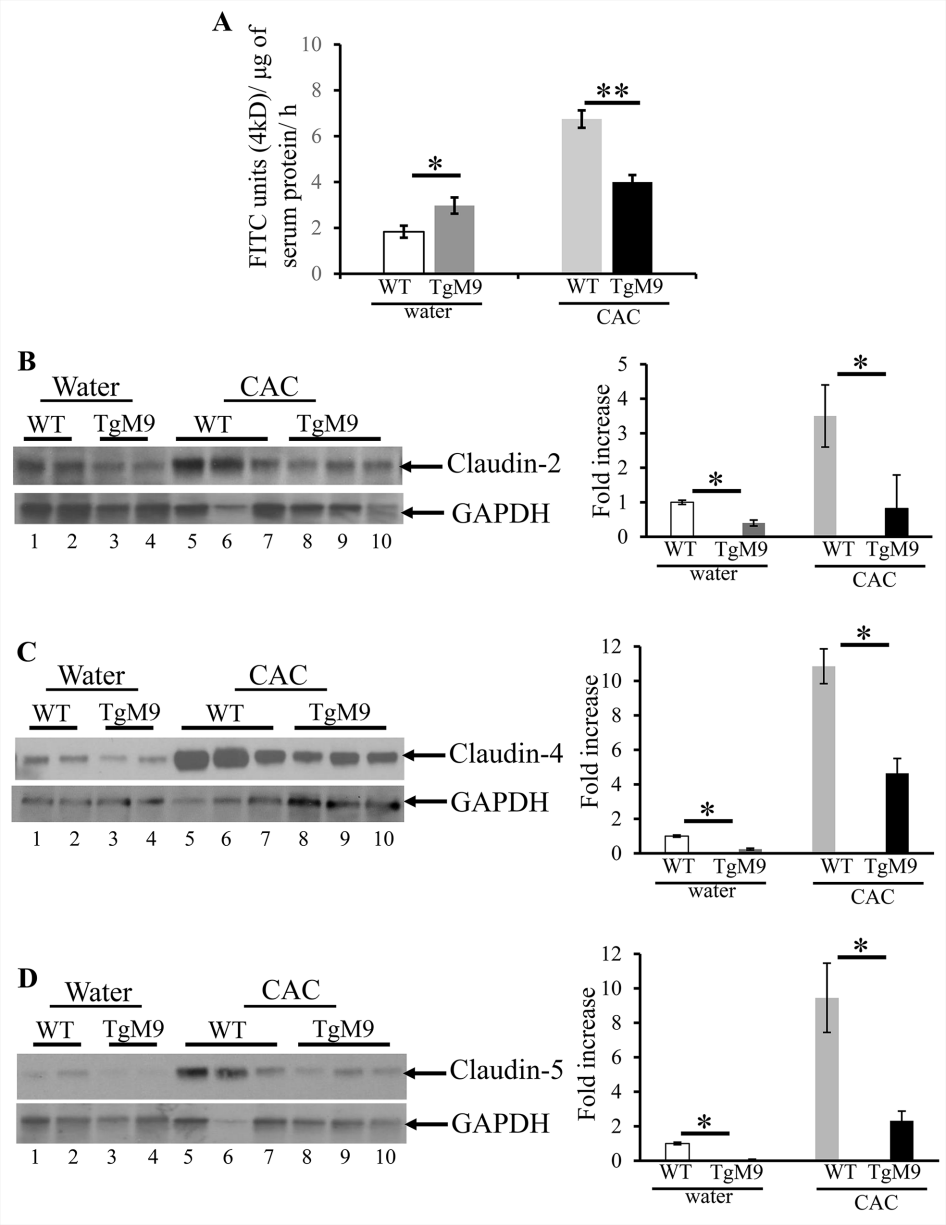
MMP9 maintains colonic epithelial barrier function and TJ assembly in CAC *In vivo* permeability was measured in TgM9 and WT mice treated with water or 3 cycles of 3% DSS to assess the barrier function between the lumen and the mucosal epithelial lining by using 4kD FITC dextran molecule. **(A)** Flux of 4kD FITC units/μg of serum protein. Each bar represents mean ± S.E., *p< 0.05, **p< 0.005. Paracellular permeability and TJ integrity of colonic epithelium was assessed by performing WBs (25ul/lane) using whole cell lysates of colon mucosal stripping and probed with **(B)** Claudin-2 **(C)** Claudin-4 and **(D)** Claudin-5. The loading control for each blot was GAPDH. Each blot was a representation of three individual experiments. Densitometry evaluations of the WB is represented by the adjacent bar graph and each bar represents mean ± S.E., *p< 0.05.

Epithelial barrier integrity is critical in regulating homeostasis between the lumen and the mucosal epithelial lining [30]. In CAC, inflammatory and immune responses can compromise the epithelial integrity resulting in the disease advancement. TJs, which control the solute flux between the lumen and the mucosal epithelial lining, are made up of transmembrane proteins such as claudins, occludins and peripheral proteins zonula occludens [30]. We investigated the role of MMP9 in regulating the barrier function by measuring the *in vivo* permeability (see Methods section) using 4kD fluorescein isothiocyanate (FITC) dextran molecule. We observed that TgM9 mice displayed efficient barrier function (4.0±0.31 flux of 4kD FITC units/μg of serum protein) compared to WT mice in CAC (6.8±0.38 flux of 4kD FITC units/μg of serum protein) as shown in Figure 3A. However, TgM9 had leaky epithelium (3.0±0.35 flux of 4kD FITC units/μg of serum protein) compared to WT mice (1.8±0.26 flux of 4kD FITC units/μg of serum protein) (Figure 3A) both exposed to water/ without CAC. Claudin-2, claudin-4 and claudin-5 are the most abundant TJ proteins which regulate the paracellular permeability during colonic inflammation [31]. We performed western blot (WB) using the whole cell lysates of the mucosal stripping of the colons and observed that TgM9 exhibited significantly decreased expression of claudin-2, claudin-4 and claudin-5 (Figure 3B-3D respectively) as represented by lanes 8-10, compared to WT mice (lanes 5-7) in CAC. We also observed a subtle decrease in the expression of claudins-2, -4 and -5 among TgM9 mice (Figure 3B-3D; lanes 3-4, respectively) compared to WT mice (Figure 3B-3D; lanes 1-2, respectively) both exposed to water/ without CAC. These data together indicate that MMP9 mediates epithelial integrity by maintaining the barrier function and the TJ integrity in CAC.

### MMP9 maintains mucosal layer in CAC

In the colon, the mucus is firmly adhered to the epithelial cells and harbors the microbiota. The mucus is organized in two layers: the inner one and an outer “not so firm/-bit loose” layer [32]. MUC2, mucin protein of the colon mucus, provides nutrients to microbiota and its O-glycan serves as attachment site for them. There are three trefoil factors (TFF) which are prevalent in humans as the secretory products of mucous epithelia. Among the three TFFs, TFF3 is generated in intestinal goblet cells in combination with MUC2 [33].

**Figure 4:**
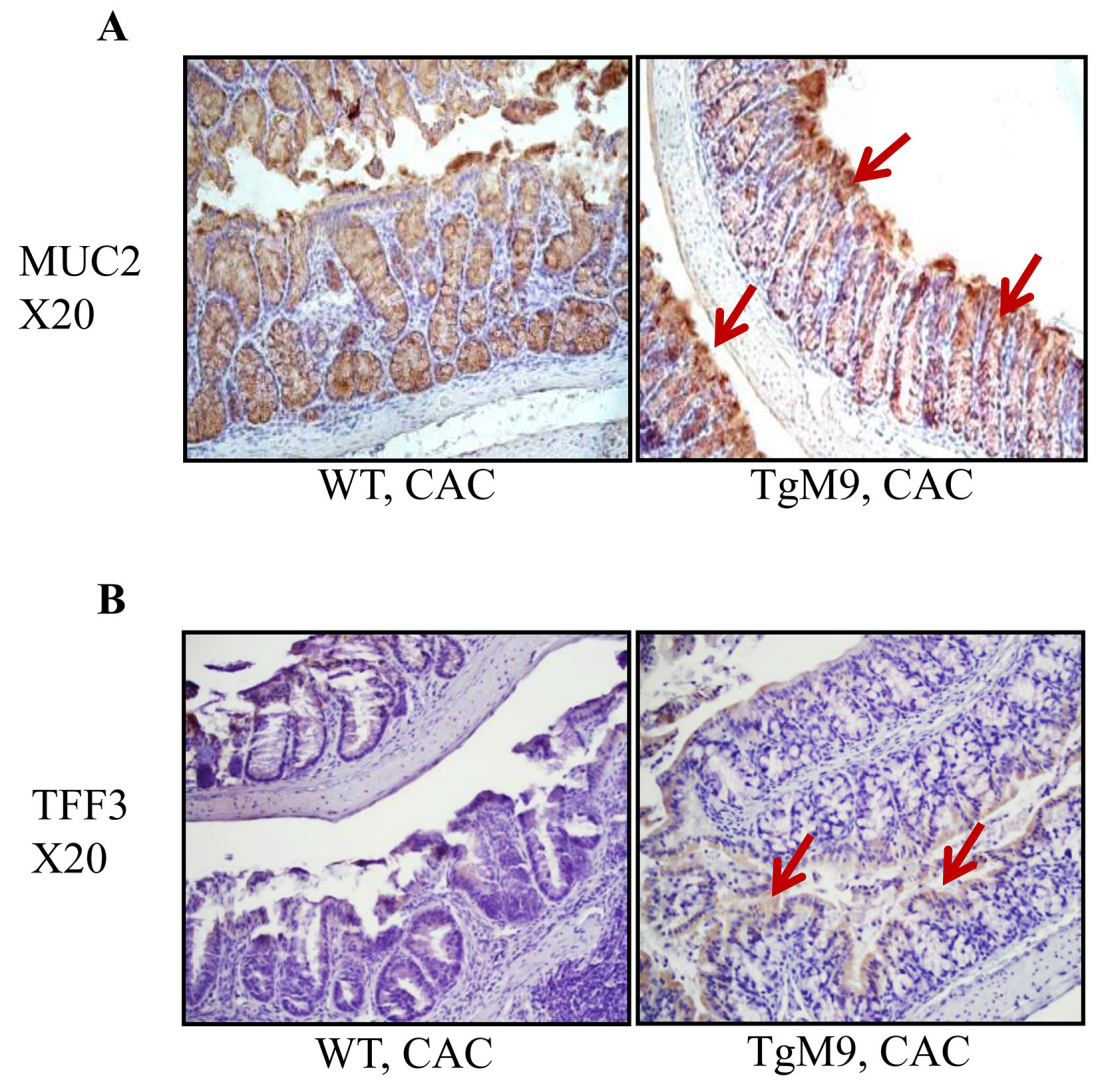
MMP9 maintains mucin levels in CAC Immunostaining of the Swiss rolls from WT and TgM9 mice treated with 3 cycles of 3% DSS was performed to investigate the integrity of mucosal-epithelial layer. **(A)** MUC2 and **(B)** TFF3 expression among TgM9 is indicated by the red arrows. Microscopic images were taken at X20 magnification.

Therefore, we investigated how MMP9 regulates MUC2 levels by performing immunostaining of the Swiss roll of mice colons (as described in the Methods section). We observed that TgM9 had increased MUC2 expression compared to WT mice in CAC, as indicated by brown colored staining in Figure 4A (shown by red arrows). We also observed that there was an increased expression of TFF3 among TgM9 mice compared to WT mice as represented by brown colored staining in Figure 4B (shown by red arrows).

However, we observed that TgM9 mice had decreased expressions of MUC2 as well as TFF3 compared to WT mice, as represented by brown colored staining (Supplementary Figure 1) when both of the groups were exposed to water/ without CAC.

### MMP9 overexpression is associated with decreased paracellular permeability

**Figure 5:**
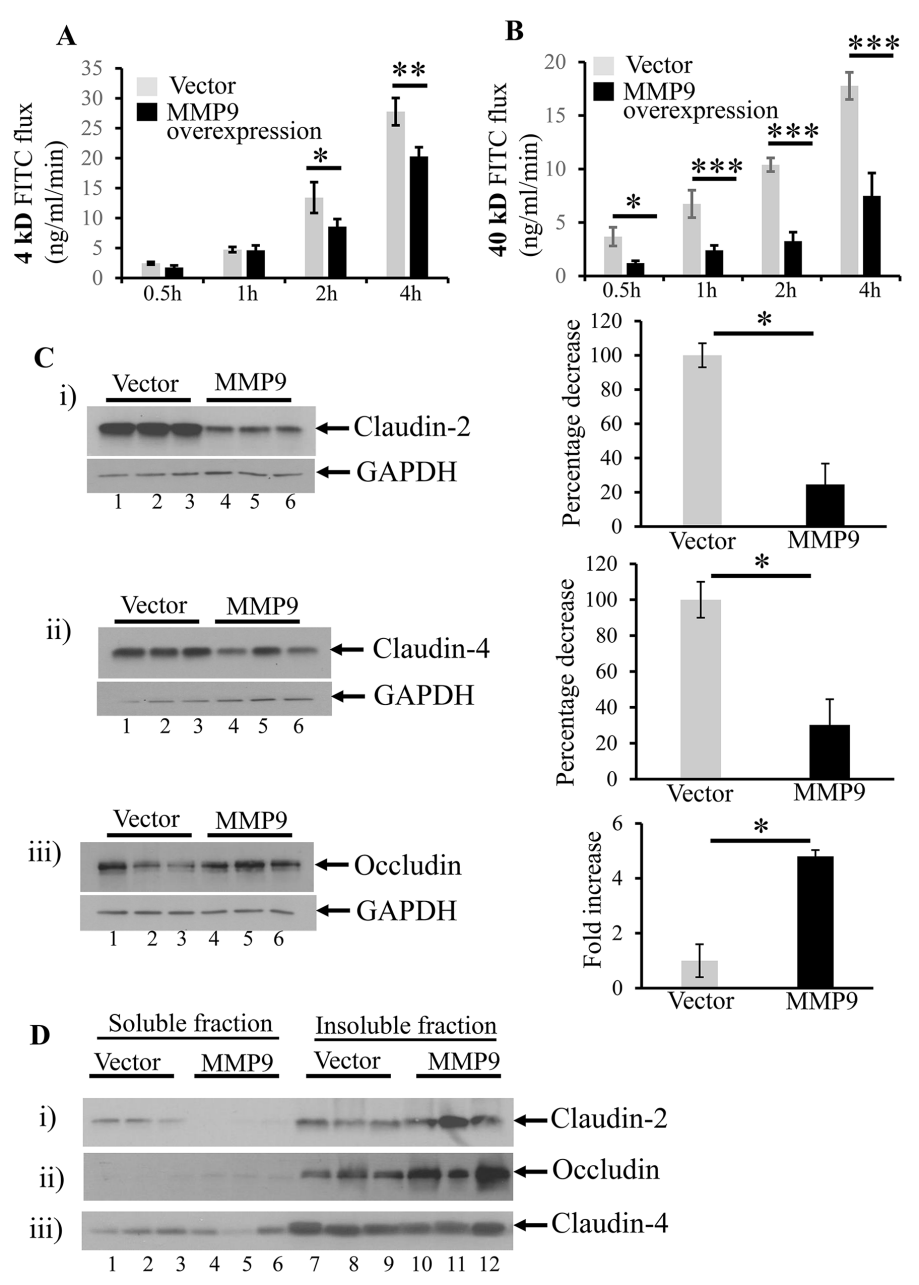
MMP9 overexpression is associated with decreased paracellular permeability *In vitro* model of stably transfected human intestinal epithelial cells CaCo2BBE overexpressing MMP9 were used to assess paracellular permeability at 0.5 hour, 1 hour, 2 hours and 4 hours by using 4kD and 40kD FITC dextran molecule. **(A)** Flux of 4kD FITC ng/ml/min. **(B)** Flux of 40kD FITC ng/ml/min. Each bar represents mean ± S.E., *p< 0.05, **p< 0.005, ***p< 0.0005. The organization of TJs was assessed by performing WBs (30μg/lane) with whole cell lysates probed with **(C i-iii)** Claudin-2, Claudin-4 and Occludin respectively. The loading control for each blot was GAPDH. Each blot was a representation of three individual experiments. Densitometry evaluations of the WB is represented by the adjacent bar graph and each bar represents mean ± S.E., *p< 0.05. The integrity of TJs were studied by performing WBs (10μg/lane) for Triton X-100 soluble versus Triton X-100 insoluble fraction **(D i-iii)** Claudin-2, Occludin and Claudin-4 respectively.

We used the *in vitro* model of stably transfected human intestinal epithelial cells CaCo2BBE cells overexpressing MMP9 to validate the *in vivo* data. We have previously published the efficiency of the stably transfected CaCo2BBE cells with and without MMP9 [34]. Figure 5A shows that overexpression of MMP9 in CaCo2BBE was associated with significantly decreased paracellular permeability of 4kD FITC dextran at 2 hours (8.58±1.27 FITC flux in ng/ml/min) and 4 hours (20.29±1.54 FITC flux in ng/ml/min) compared to vector control (13.43±2.57 and 27.77±2.27 FITC fluxes in ng/ml/min respectively). Likewise, CaCo2BBE cells overexpressing MMP9 also displayed (Figure 5B) a significant decrease in the paracellular permeability of 40kD FITC flux in ng/ml/min at 30 minutes (1.21±0.21), 1 hour (2.40±0.46), 2 hours (3.2±0.84) and 4 hours (7.49±2.14) compared to vector control (3.68±0.87, 6.73±1.28, 10.4±0.64 and 17.77±1.27 FITC fluxes in ng/ml/min respectively).

We also assessed the organization of TJs by performing WBs with whole cell lysates. We observed decreased expressions of claudin-2 (Figure 5C-i) and claudin-4 (Figure 5C-ii) but increased expression of occludin (Figure 5C-iii) among MMP9 overexpressing CaCo2BBE cells (lanes 4-6) compared to the vector control (lanes 1-3).

We also investigated the expressions of membrane bound claudins and occludin in the Triton X-100 insoluble fraction versus Triton X-100 soluble fraction to understand the integrity of TJ assembly. We observed that MMP9 expression (lanes 10-12) was associated with increased levels of membrane bound claudin-2 (Figure 5D-i) and occludin (Figure 5D-ii) compared to their presence in the cytoplasmic (Triton X-100) soluble fractions (lanes 4-6). Interestingly, there was no change in the expression levels of membrane bound claudin-4 (lanes 10-11, Triton X-100 insoluble fraction) versus cytoplasmic claudin-4 (lanes 4-6) as represented by WB in Figure 5D-iii. We also observed that vector without the overexpression of MMP9 showed increased cytoplasmic claudin-2 in Triton X-100 soluble fractions (lanes 1-3, Figure 5D-i compared to MMP9 overexpressing cells lanes 4-6, Figure 5D-i). However occludin was less in Triton X-100 insoluble fraction of vector control (lanes 7-9, Figure 5D-ii) compared to MMP9 overexpressing cells (lanes 10-12, Figure 5D-ii).

**Figure 6:**
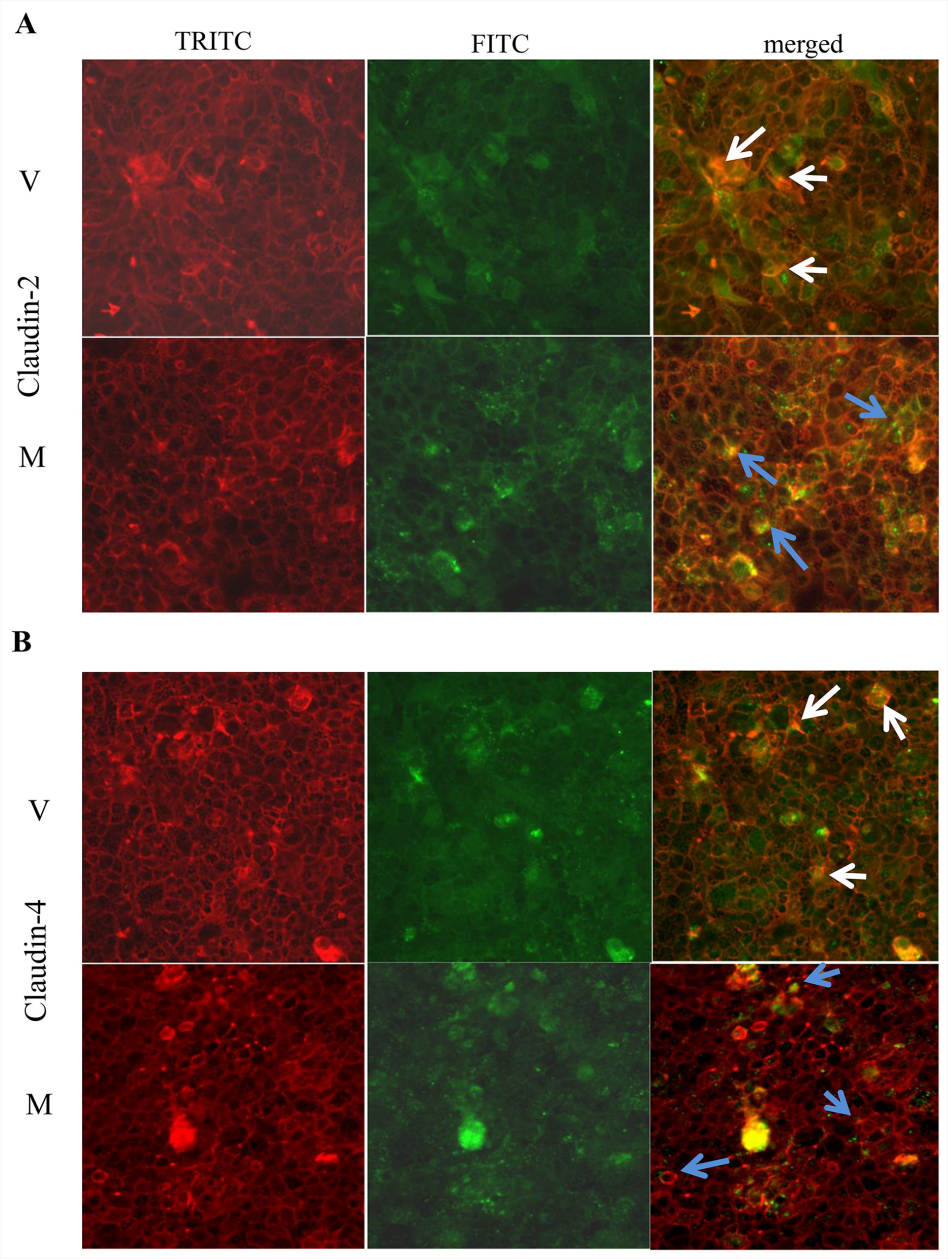
MMP9 overexpression preserves TJ integrity Immunofluorescence staining was performed using *in vitro* model CaCo2BBE cells overexpressing MMP9 to investigate TJ distribution. Distribution of **(A)** Claudin-2 and **(B)** Claudin-4 are indicated by blue arrows among MMP9 overexpressing CaCo2BBE cells and by white arrows among vector control.

Immunofluorescence staining showed that claudin-2 (Figure 6A) and claudin-4 (Figure 6B) were more confined to the junctions (as indicated by blue arrows) among MMP9 overexpressing CaCo2BBE cells compared to the vector control. Vector control showed the irregular distribution of claudin-2 and claudin-4 (Figure 6A and 6B respectively) as indicated by white arrows.

These data together imply that MMP9 expression is associated with tighter epithelial barrier by retaining the TJ assembly.

### MMP9 activates EGFR1 signaling

**Figure 7:**
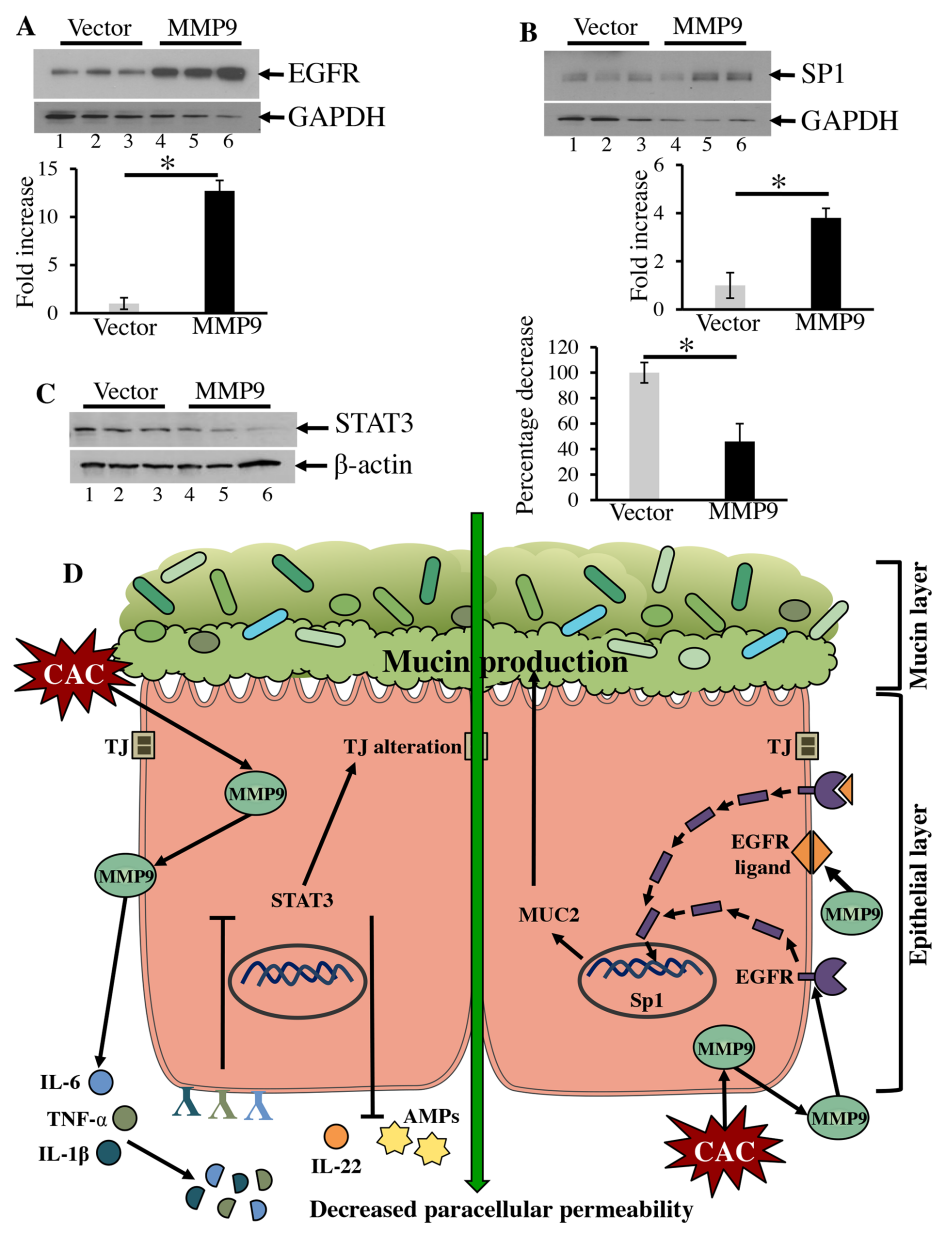
MMP9 activates EGFR1 signaling The regulation of MUC2 was investigated by using CaCo2BBE cells overexpressing MMP9 and performing WBs (25μg/lane) using whole cell lysates probed with **(A)** anti-EGFR, **(B)** anti-Sp1 and **(C)** anti-STAT3. The loading control for the each blot were GAPDH or β-actin. Each blot was a representation of three individual experiments. Densitometry evaluations of the WB is represented by the adjacent bar graph with each bar represents mean ± S.E., *p< 0.05. **(D)** Schematic representation of MMP9 mediated maintenance of epithelial-mucosal integrity in CAC. In CAC, MMP9 cleaves pro-inflammatory cytokines which fail to bind their respective receptors and may downregulate STAT3 signaling. Downregulated STAT3 supports TJ integrity in CAC as well as contribute to the downregulation of IL-22 and AMPs levels. This leads to epithelial integrity and hence the preservence of microbiome. MMP9 being a secretory proteinase can activate EGFR signaling by cleaving EGF family ligands or by direct cleaving of the EGFR receptor. Cleaved or cytoplasmic EGFR directly or through some downstream signaling pathway(s) can translocate to the nucleus to activate the nuclear transcription factor Sp1. *Sp1* promoter shares homology with *MUC2* promoter and therefore stimulate MUC2 expression which rebuilds colonic mucus layer and promotes microbiota adherence.

EGFR signaling is well documented in proliferation and development of epithelial cells in multiple organs such as skin, kidney, brain, and GI tract [35, 36]. EGFR is activated either by ligand binding (EGF family ligands) or transactivation mechanisms (by G protein coupled receptors) initiating several downstream targets. Other than participating in canonical cell-surface activities, EGFR can also localize to the nucleus and regulate different transcriptional factors associated with cell growth and proliferation [35]. The *MUC2* promoter of MUC2 mucin protein is also known to be regulated by the transcription factor specificity protein 1 (Sp1), whose activity is regulated by EGFR signaling [37]. Figure 7A shows that MMP9 overexpressing CaCo2BBE cells (Lanes 4-6) had an increased expression of EGFR1 compared to vector control (lanes 1-3). Figure 7B displays that the MMP9 expression (lanes 4-6) was also associated with an increased expression of nuclear transcription factor SP1 compared to vector control (lanes 1-3). We also observed a significant decrease in STAT3 expression among MMP9 overexpressing cells (Figure 7C, lanes 4-6) compared to vector control (Figure 7C, lanes 1-3).

These data together imply that MMP9, being a secretory proteinase, activates transcellular EGFR1 directly through nuclear translocation or via some other signaling cascade, which then activates the nuclear transcription factor Sp1 to maintain mucin levels (Figure 7D).

## DISCUSSION

Inflammation was thought to be an effect of carcinoma, but recently it has gathered much attention as it is considered to be one of the causative agents of GI cancer [23, 38]. CAC progresses through low to high-grade dysplasia and it is different than CRC progression. CRC advances through progressive stages of adenomas. There are significant gaps in understanding the mechanism of the CAC due to the following two reasons: i) Alteration in gut lumen agents such as microbiota population, expression of inflammatory cytokines, and reactive oxygen species (ROS) due to chronic inflammation in CAC. ii) The autoantigens thus expressed by inflamed/dysplastic cells stimulate non-specific events, initiating cellular destruction of epithelium. Chronic inflammation causes sustained proliferation favoring an environment rich in inflammatory cells, chemokines/cytokines, and AMPs, which contribute in nurturing the tumor microenvironment through the “extrinsic mechanistic pathway” [28, 29, 39]. These mediators regulate the microbiota population, immune homeostasis, and act as checkpoints for the microenvironment desired for tumor progression.

In this study, we have observed that in CAC, MMP9 expression in colonic epithelium preserves the microbiota as evident by increased mRNA levels of the *16SrRNA*. MMP9 favors the higher ratio of *Bacteroidetes* and *Fermicutes* as well as increased mRNA levels of mucolytic bacteria *A. muciniphila* in CAC. We also observed increased mRNA levels of *REG3* family (*REG3-a* and *REG3-b*) and AMPs, while a decrease in mRNA levels of *REG3-g* and *S100A8* among TgM9 mice. We also observed that MMP9 expression is associated with an increase in mRNA levels of pro-inflammatory cytokines such as IL-6, TNF-α, IL1β and IFN-γ but a decrease in mRNA levels of inflammatory cytokine IL-22. TgM9 also mice exhibited efficient barrier function and decreased expressions of claudins-2, 4, and -5 compared to WT mice in CAC. The i*n vitro* data showed higher expression of most of the TJs in the Triton X-100 insoluble fraction of MMP9 overexpressing cells. This suggests that MMP9 maintains the TJ proteins integrity in CAC. TgM9 mice showed increased MUC2 and TFF3 levels compared to WT mice in CAC. *In vitro* data indicated that MMP9 expression is associated with increased protein levels of EGFR and Sp1. It utilizes EGFR-Sp1 signaling to maintain mucin levels as an alternate tumor suppressive pathway in CAC. Altered microbiome and barriers are two intertwined events. However depending on the context, either of them can precede over other. Our study suggests that in CAC, activation of MMP9 prevents the loss of tight junctions resulting in the maintenance of efficient epithelial barrier favoring epithelial cell homeostasis which helps in maintaining microbial population in colonic epithelial mucosa. These results together imply that MMP9 mediates its protective role in CAC by maintaining epithelial-mucosal integrity and; therefore, acts as a tumor suppressor.

Due to the production of inflammatory metabolites, chronic inflammation dysregulates the host immune system leading to the misbalance of the microbial population in colon. A direct association between MMP9 expression and increased *16SrRNA* mRNA levels reflects the diversity and the richness of microbiome and implies the reduced risk of CAC progression. However, it is also important to identify the associated signaling pathway contributing to the preservation of the microbiome. The higher mRNA ratio of *Bacteroidetes* and *Fermicutes* and increased *A. muciniphila* mRNA levels, among TgM9 mice in CAC, suggests that MMP9 mediated epithelial and immune homeostasis supports the good bacterial populations in the disease. A lower ratio of *Bacteroidetes* over *Fermicutes* have also been observed in several inflammatory conditions such as inflammatory bowel syndrome (IBS), IBD, obesity, and type 2 diabetes [40, 41]. The positive association between MMP9 and abundance of *A. muciniphila* in CAC indicates that the decreased inflammation could be due to the efficient colonic barrier function and immune homeostasis.

It is known that AMPs are the small peptides which contribute to the front line of defense as effector molecules to maintain innate immune system [25, 42, 43]. Therefore, they are critical in regulating microbiota dysbiosis during chronic colonic inflammation and colorectal cancer. Among AMPs, REG3 and S100A families are important in context of inflammatory conditions [27, 44–46]. MMP9 mediated significant increase in REG3-a, -b, while decrease in REG3-g and S100A8 in CAC, suggest that the expression of multiple REG members at the same site might act as a compensatory mechanism. Therefore, further research is required to investigate the direct correlation between AMPs (REG3 and S100A families) expressions and the tumor microenvironment progression in CAC.

MMP9 mediated increase in pro-inflammatory cytokines such as IL-6, IL-1β, TNF-α, IFN-γ could be the result of the proteolytic activity of the proteinase MMP9. Other researchers have also shown the same suggesting that the cleaved cytokines maintain the MMP9 levels by a positive feedback mechanism or by activating other signaling pathways [16, 47–49]. We hypothesize that (Figure 7D) due to the proteolytic activity of MMP9, the binding affinity of the truncated cytokines to their respective cellular receptors is compromised. This downregulates the STAT3 pathway (Figure 7D) [28, 50, 51]. Downregulated STAT3 pathway promotes the TJ integrity and efficient barrier function in CAC (Figure 7D) [30]. We also hypothesize that (Figure 7D) downregulated STAT3 suppresses the IL-22 levels. IL-22 has been shown to be important for mucosal wound healing in acute colitis model [52]. However, recently it has been reported that IL-22 can also promote pro-inflammatory cytokine secretion initiating the chronic inflammation [29, 53]. It has also been reported that IL-22 is important for the progression of carcinogenesis through the STAT3 pathway [54]. The low mRNA levels of REG3-g among TgM9 in CAC could also be a result of downregulated IL-22 secretion.

Our data clearly implies a direct correlation between MMP9 expression and efficient epithelial barrier function as evident by decreased paracellular permeability and reorganization of claudins (Figure 7D). Increased expressions of claudins -4 and -5 contribute significantly in increased paracellular permeability and claudin-2 plays a major role in maintaining the charge selectivity of the TJs [55]. Importance of intact mucin layer over epithelial cells as a defensive mechanism against luminal insults has been widely described in literature [32, 56, 57]. Our data showed that MMP9 mediated protection in CAC maintains epithelial integrity as well as mucosal integrity. Increased expressions of MUC2 and TFF3 proteins among TgM9 suggest that the intact mucin layer supplies nutrients to maintain the microbiota as well as protects the epithelial cell surface in chronic inflammatory environment.

Our group has previously shown that MMP9 mediates enterocyte differentiation via activation of Notch1 [34]. However in CAC, MMP9 utilizes EGFR signaling as an alternative mechanism to activate the nuclear transcription factor Sp1 to maintain mucin levels. Promoter of *Sp1* shares common regulatory elements with *MUC2* promoter [58]. This enables MMP9 to maintain the mucosal-epithelial integrity, microbiota, and immune homeostasis to protect against luminal insults in CAC.

Recently, humanized versions of synthetic peptides such as GS5745, Col-3, REGA-3G12 have been synthesized to inhibit the proteolytic or functional activity of MMP9 [59, 60]. However efficacy of these therapies is tested either by acute inflammatory models or CRC models. Unfortunately, none of them represented the CAC-chronic inflammatory model. This implies a paradox in the future use of these as treatments. In this context, it is important to mention that recently the clinical trial by Geliad with GS5745 as a therapeutic strategy to treat mild to severe UC (inflammatory condition in colon) patients was terminated for not meeting the pre-specified efficacy criteria. Extrapolation of our research to clinical practice will establish the fact that in chronic inflammatory conditions of colon direct and/or indirect silencing of MMP9 should be avoided. Our study recognizes MMP9 as a beneficial proteinase due to its protective role in CAC.

## MATERIALS AND METHODS

### Animal models

All animal procedures were in compliance with the Guide for the Care of Use of Laboratory Animals from the US Public Health Service and with approval from the Animal Care Committee of Georgia State University. As described previously [15], 10 weeks old gender matched TgM9 and their wild-type (WT) littermates of C57/B6 background were used for the study. Comprehensive characterization of TgM9 have been done and published by our group [14, 15]. Mice were maintained on a 12-hour dark-light cycle and allowed free access to no purified diet pellets and tap water.

### CAC induction

Animals were divided into two groups- one as control group, exposed to water only (without CAC) and another as experimental group with CAC induction. As described previously [15], CAC was induced among both TgM9 mice (n=30) and WT (n=30) mice by 3 cycles of dextran sodium sulfate (DSS) (MP Biomedicals, Salon, OH). Control group including both TgM9 and WT mice were exposed to drinking water only. Mice were exposed to 3% DSS (w/v) by oral administration through their drinking water ad libitum for a week followed by two weeks of recovery cycle. On day 85, the mice were sacrificed after 3rd cycle of DSS and recovery (Supplementary Figure 2). Mice were monitored for body weight, stool consistency, and stool occult blood during DSS and recovery cycles.

### Cell culture and transfection

As described previously stably transfected CaCo2BBE cell (ATCC, Manassdas, VA) with and without MMP9 [34] were used to assess the paracellular permeability and the TJ organization by WB and immunofluorescence staining. They were transfected for 72 h with a pEGFP plasmid with and without the MMP9 gene in 6 well plate. The transfected clones were selected under an antibiotic (Geneticin; GIBCO, Grand Island, NY). These transfected clones were screened for MMP9 expression and the three highest MMP9 expressing clones were selected for CaCo2BBE cell line and then they were sorted via flow cytometry (BD Biosciences).

### RNA extraction and QPCR

Colons were harvested from the TgM9 and WT littermate mice with and without CAC. These colons were then gently flushed with phosophate buffered saline (PBS) to remove feces. Total RNA was extracted from colonic tissues using the RNeasy mini Kit (Qiagen, Valencia, CA) according to the manufacturer’s instructions. Yield and quality of RNA were verified with a Synergy 2 plate reader (BioTek, Winooski, VT). Complementary DNA was generated from the earlier-described total RNA isolated using the Maxima first-strand complementary DNA synthesis kit (Thermo Scientific, Lafayette, CO). mRNA expression was quantified by quantitative real-time reverse-transcription polymerase chain reaction using Maxima SYBR green/ROX (6-carboxyl-X-rhodamine) quantitative polymerase chain reaction Master Mix (Thermo Scientific) and the following sense and antisense primers: *Firmicutes*, *Bacteroidete*s, *A. muciniphila*, *REG3-a*, *REG3-b*, *REG3-g*, *S100A8*, *IL-22*, *IL-6*, *IL-1β*, *TNF-α*, and *IFN-γ* (Supplementary Table 1).

### *In vivo* and *in vitro* permeability

*In vivo* permeability assay was performed to assess barrier function using a FITC-labeled dextran method as described [14, 61]. Briefly, 8-weeks-old WT and TgM9 mice were used. Food and water were withdrawn for 4 h, and mice were gavaged with permeability tracer (60 mg/100 g body weight of 4kD FITC-labeled dextran) (Sigma-Aldrich Corp, St. Loius, MO). Serum was collected retro-orbitally 4 h after the gavage, and fluorescence intensity of each sample was measured (excitation, 492 nm; emission, 525 nm; Cytofluor 2300; EMD Millipore, Billerica, MA), and FITC-dextran concentrations were determined from standard curves generated by serial dilution of FITC-dextran. Permeability was calculated by linear regression of sample fluorescence (Excel, Microsoft Office).

CaCo2BBE cells transfected with or without MMP9 were allowed to grow to 100% confluency on filters (Corning, Tewksbury, MA). For *in vitro* permeability assays, cells were treated with 250 ng/ml FITC-dextran (MW 4 kDa and 40 kDa; Sigma-Aldrich Corp.). The apical and basolateral reservoirs were sampled at the indicated time points and FITC-dextran concentration was quantified via spectrofluorimetry (λex=492 nm, λem=525 nm). FITC-dextran concentrations were determined from standard curves generated by serial dilution of FITC-dextran.

### Western blot

As described previously [10], for WB analysis, colonic mucosal stripping was obtained from the TgM9 and WT mice (n=20 per group) with and without CAC. *In vivo* WB analysis was performed with 30μg/ well of cell lysates. WB analysis for *in vitro* model was performed using whole cell lysates (30μg/ well) of CaCo2BBE cells with and without MMP9. 10^6^ cells of CaCo2BBE were grown on 6 well plate for 100% confluency. As described by Bazzoni *et al* [62], we collected Triton X-100 soluble and insoluble fraction of CaCo2BBE cells with and without MMP9 to assess TJ integrity and 10μg/ well was used for WB analysis. The antibodies used were anti-MMP9 (Abcam, Cambridge, MA), anti-EGFR (Cell Signaling, Beverly, MA), anti-Claudin-2 (Life Technologies, Rockford, IL), anti-Claudin-4 (Invitrogen, Rockford, IL), anti-Claudin-5 (Invitrogen), anti-TFF3 (Cloud-Clone Corp., Katy, TX), anti-Sp1 (Upstate Cell Signaling Solutions, Lake Placid, NY), anti-Occludin (Invitrogen), anti-STAT3 (Cell Signaling). Goat anti-mouse secondary antibody (Bio-Rad, Hercules, CA) or goat anti-rabbit secondary antibody (Bio-Rad) were used. Densitometry graphs were generated by using image acquisition and analysis software by VisionWorksLS Analysis Software (UVP, Upland, CA).

### Immunostaining

Colons were fixed and embedded in paraffin. For MUC2 and TFF3, sections were deparaffinized and incubated with 0.3% hydrogen peroxide. Sections were washed with 1X PBS and then incubated with sodium citrate buffer. Sections were then cooked in a pressure cooker for 10 minutes and washed after. Sections were blocked with goat serum antibody for 45 mins. After this pre-treatment, sections were incubated with MUC2 (Santa Cruz Biotechnology, Dallas, TX) and TFF3 individually at 4°C overnight. Sections were then washed and incubated with the appropriate biotinylated secondary antibodies for 30 minutes at room temperature. Color development was achieved by using ABC kit (Vector Laboratories, Blurlingame, CA). After washing, sections were counterstained with hematoxylin, dehydrated, and sealed.

### Immunofluorescence staining

CaCo2BBE cells grown in Tissue-Tek chambers (Thermo Fischer Scientific, Waltham, MA) were fixed with 3.7% paraformaldehyde for 20 minutes at room temperature. Cells were washed with 1X PBS (with Tween 20) and then blocked with 3% BSA in 1X PBS for 1 hour. Cells were stained with phalloidin-Tetramethylrhodamine B isothiocyanate (Sigma-Aldrich Corp) for 1 hour at room temperature. After washing with 1X PBS (with Tween 20), cells were incubated overnight at 4°C with Claudin-2 (Life Technologies) and Claudin-4 (Invitrogen). After washing, cells were incubated with their appropriate FITC secondary antibody for 1 hour at room temperature. After washing, the cells were mounted with ProLong Antifade mounting medium (Thermo Fischer Scientific) and were analyzed using the fluorescent microscope.

### Statistics

As described previously [15], data are presented as means ± SE. Groups were compared by Student’s t-test. P values <0.05 was considered statistically significant.

## SUPPLEMENTARY MATERIALS FIGURES AND TABLE


