# Supplementary material for: Matrix metalloproteinase MMP9 maintains epithelial barrier function and preserves mucosal lining in colitis associated cancer
Source: Oncotarget. 2017 Oct 17;8(55):94650–65. doi: 10.18632/oncotarget.21841 (PMC5706902; doi:10.18632/oncotarget.21841)
Supplement: Supplementary file 1 [file oncotarget-08-94650-s001.pdf]

Matrix metalloproteinase MMP9 maintains epithelial barrier function and preserves mucosal lining in colitis associated cancer

SUPPLEMENTARY MATERIALS

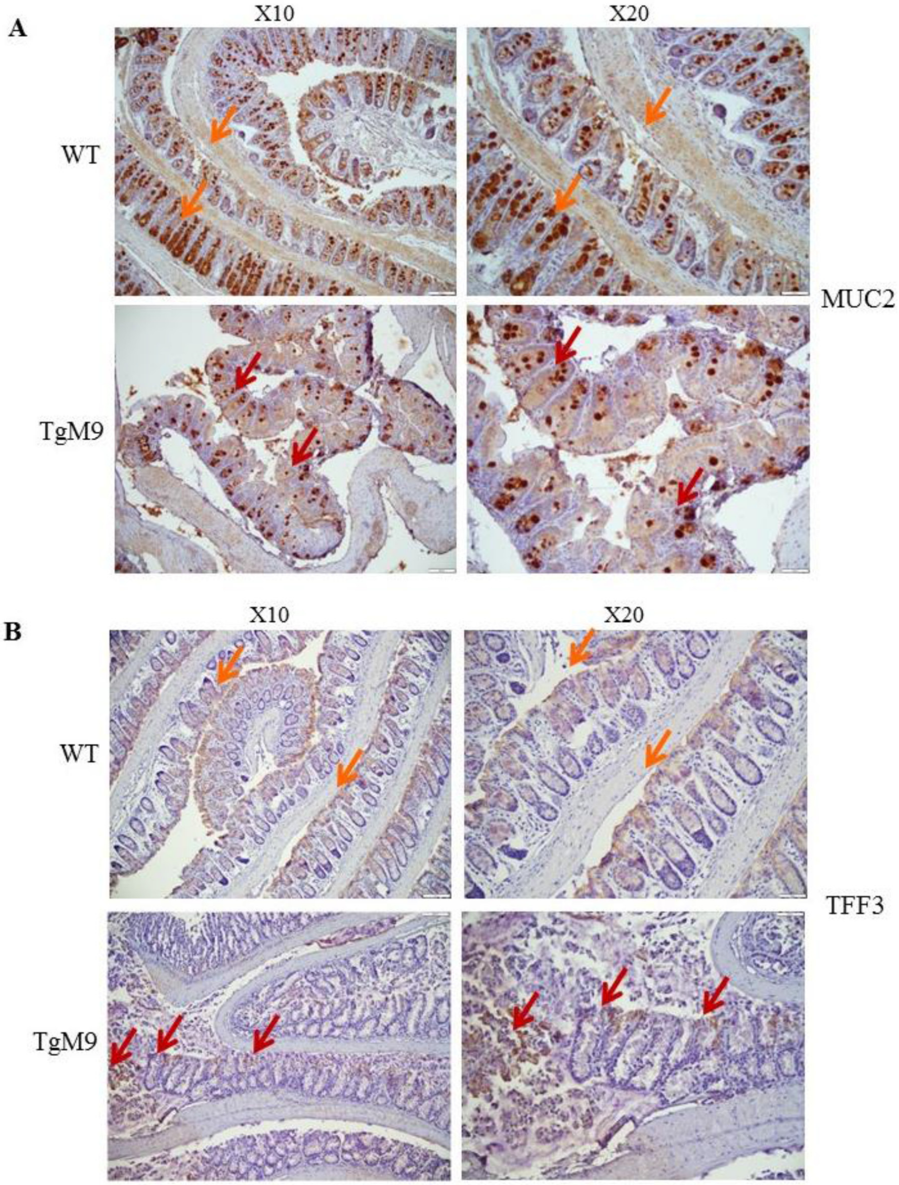

Supplementary Figure 1: MUC2 and TFF3 expressions in TgM9 mice and WTs without CAC. Immunostaining of the Swiss roll of the colons of TgM9 and WT mice (n=10 each group) with **(A)** anti-MUC2; **(B)** anti-TFF3. Red arrows indicate brown staining among TgM9 mice and orange arrows indicate brown staining among WT littermates exposed to water/ without CAC. Images are representative of two experiments.

Supplementary Table 1: Sequences of the QPCR primers used in the study

Gene	Forward (5'-3')	Reverse (5'-3')
Firmicutes	GGCAGCAGTRGGGAATCTTC	ACACYTAGYACTCATCGTT
Bacteroidetes	GGARCATGTGGTTTAATTCGATGAT	AGCTGACGACAACCATGCAG
A. Muciniphila	CAGCACGTGAAGGTGGGGAC	CCTTGCGGTTGGCTTCAGAT
Reg3-a	GGCACCGAGCCCAATG	GGATTTCTCTCCCATGCAAAGT
Reg3-b	ATACCCTCCGCACGCATTAGTT	AGG CCAGTTCTG CATCAAACCA
Reg3-g	TTCCTGTCCTCCATGATCAAAA	CATCCACCTCTGTTGGGTTCA
S100A8	CATCCACCTCTGTTGGGTTCA	CATCCACCTCTGTTGGGTTCA
IL-22	GTCAACCGCACCTTTATGCT	GTTGAGCACCTGCTTCATCA
IL-6	ACAAGTCGGAGGCTTAATTACACAT	TTGCCATTGCACAACTCTTTTC
IL-1ß	TCGCTCAGGGTCACAAGAAA	CATCAGAGGCAAGGAGGAAAAC
TNF-α	AGGCTGCCCCGACTACGT	GACTTTCTCCTGGTATGAGATAGCAAA
IFN γ	CAGCAACAGCAAGGCGAAA	CTGGACCTGTGGGTTGTTGAC
16s rRNA	AGAGTTTGATCCTGGCTCAG	CTGCTGCCTCCCGTAGGAGT

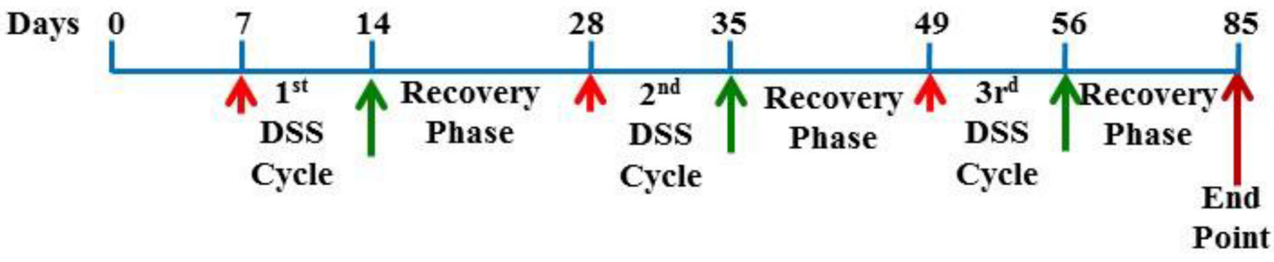

Supplementary Figure 2: Schematic of CAC model used for the study. CAC was induced by three cycles of DSS (1 week long). Each DSS cycle was followed by two weeks of recovery cycle. End point of the experiment was day 85. Red arrows represent the start point of DSS cycle and green arrows represent the start point of recovery cycle.
